# Evaluation and Assessment of Trivalent and Hexavalent Chromium on *Avena sativa* and Soil Enzymes

**DOI:** 10.3390/molecules28124693

**Published:** 2023-06-10

**Authors:** Edyta Boros-Lajszner, Jadwiga Wyszkowska, Jan Kucharski

**Affiliations:** Department of Soil Science and Microbiology, University of Warmia and Mazury in Olsztyn, Plac Łódzki 3, 10-727 Olsztyn, Poland; edyta.boros@uwm.edu.pl (E.B.-L.); jan.kucharski@uwm.edu.pl (J.K.)

**Keywords:** Cr(III), Cr(VI), plant tolerance to chromium, chromium translocation in the plant, soil biochemical properties

## Abstract

Chromium (Cr) can exist in several oxidation states, but the two most stable forms—Cr(III) and Cr(VI)—have completely different biochemical characteristics. The aim of the present study was to evaluate how soil contamination with Cr(III) and Cr(VI) in the presence of Na_2_EDTA affects *Avena sativa* L. biomass; assess the remediation capacity of *Avena sativa* L. based on its tolerance index, translocation factor, and chromium accumulation; and investigate how these chromium species affect the soil enzyme activity and physicochemical properties of soil. This study consisted of a pot experiment divided into two groups: non-amended and amended with Na_2_EDTA. The Cr(III)- and Cr(VI)-contaminated soil samples were prepared in doses of 0, 5, 10, 20, and 40 mg Cr kg^−1^ d.m. soil. The negative effect of chromium manifested as a decreased biomass of *Avena sativa* L. (aboveground parts and roots). Cr(VI) proved to be more toxic than Cr(III). The tolerance indices (TI) showed that *Avena sativa* L. tolerates Cr(III) contamination better than Cr(VI) contamination. The translocation values for Cr(III) were much lower than for Cr(VI). *Avena sativa* L. proved to be of little use for the phytoextraction of chromium from soil. Dehydrogenases were the enzymes which were the most sensitive to soil contamination with Cr(III) and Cr(VI). Conversely, the catalase level was observed to be the least sensitive. Na_2_EDTA exacerbated the negative effects of Cr(III) and Cr(VI) on the growth and development of *Avena sativa* L. and soil enzyme activity.

## 1. Introduction

Chromium is one of the transition metals, and is found in group VI B of the periodic table. It occurs in several oxidation states, the most common and stable of which are Cr (III) and Cr (VI), which differ in their chemical characteristics [1,2,3,4]. Chromium (III) exists in the following species: Cr^3+^, Cr(OH)_2_^+^, Cr(OH)_3_, Cr(OH)_4_^−^, and Cr(OH)_5_^2−^, which may occur in soil or water. Chromium (III) readily combines with oxygen to form hydroxides, sulfates, and chelate organic bonds [5]. Cr(III) can also be oxidized into Cr(VI) in high-redox soils [3,6]. These properties of chromium (III) translate to a low mobility and make it significantly less bioavailable and toxic than chromium (VI) [7,8]. The primary chromium (VI) species are CrO_4_^2−^, HCrO_4_^−^, and Cr_2_O_7_^2−^ anions, namely K₂CrO₄ and K_2_Cr_2_O_7_ [1,9]. Chromium (VI) is a potent oxidant, and can be reduced to Cr(III) in the presence of organics. The more acidic the environment, the more quickly the reduction occurs [2]. Chromium (VI) is pathogenic to humans [10,11,12], animals [13], plants [14], and microorganisms [15]. Chromium was chosen for this study due to it being one of the most toxic metal pollutants [16,17].

In plants, the toxic effects of Cr manifest as delayed seed germination, root damage and reduced root growth, reduced biomass, reduced plant height, impaired photosynthesis, membrane damage, leaf chlorosis, necrosis, low grain yield, and, ultimately, plant death [18]. Chromium is a fairly active metal and readily reacts with environmental oxygen. Trivalent and hexavalent chromium are the most stable forms of Cr in nature. In addition, Cr(VI) exhibits higher toxicity than Cr(III) due to its higher solubility and mobility in the aqueous system [19]. Both valence states of Cr, i.e., Cr(III) and Cr(VI), are taken up by plants [10]. Cr(VI) is actively taken up into plant cells by sulfate carriers [20]. On the other hand, Cr(III) enters passively through plant cell wall cation exchange sites [21]. In addition, carboxylic acids which are present in root secretions facilitate the solubilization of Cr and, thus, its uptake by plants [22].

Chromium is released naturally in the environment through rock and soil erosion, as well as by volcanic eruptions [23,24]. Its anthropogenic sources include steelmaking, papermaking, textile manufacturing, fertilizer production, pesticide production, galvanization, tanning, pigment manufacturing, nuclear weapon production, and the electronic industry [7,24,25,26,27,28]. Global chromium production increased from 23.7 to 41 million tonnes during the period from 2010 to 2021. Leading chromium producers include South Africa, India, Kazakhstan, and China [29]. The total chromium emissions in the European Union amounted to 296 tonnes in 2019, of which Poland accounted for 36 tonnes [30]. Chromium—released into the atmosphere as fly ash from CHP plants and other industrial facilities—can settle on plants and soils around the emission source or be transported by wind over long distances (depending on the size of the particles), causing plant and soil pollution [24].

Phytoextraction is a technique used to effectively remove chromium from contaminated soils by harnessing hyperaccumulator plants, which can collect and accumulate heavy metals in their aboveground parts at levels 100 times higher than other plants [31,32]. Phytoextraction can be bolstered by amending the soil with chelating agents, which can desorb metals and increase their uptake through the roots of plants [33]. EDTA (ethylenediaminetetraacetic acid) is the most effective, most popular, and a relatively stable chelator [34,35,36]. An important application of EDTA is in fixing the ions of various metals, for example bismuth, chromium (III), zinc, zirconium, aluminum, cadmium, cobalt, magnesium, copper, nickel, lead, thorium, vanadium, and iron (III), by forming stable and soluble chelate complexes [37,38,39,40]. The chelation capacity of EDTA is strong enough to even form complexes with alkaline earth metals [41]. The most commonly used chemical compound in phytoextraction is the disodium salt of ethylenediaminetetraacetic acid—Na_2_EDTA [42,43,44]. This substance, also known as Complexone III, can form chelate complexes with metal ions when dissolved in water [45]. Na_2_EDTA has been the subject of pot experiments on induced phytoextraction [35,36,43,45]. Depending on the dosage, type of metal, species of plant, and characteristics of the soil, the effectiveness of Na_2_EDTA for phytoextraction can vary considerably: from having no significant effect on metal uptake to an over 100-fold increase in phytoextraction capacity [35,36,43,45]. Na_2_EDTA has non-specific chelating properties for heavy metals such as Cr, Pb, Cu, and Zn [43,45,46,47]. *Avena sativa* L. was selected in this study for its potential usefulness in the reclamation of heavy-metal contaminated soils [48]. Due to it having a high calorific value, its grain has also been used for energy purposes, mainly for heating, especially in Scandinavian countries, with Sweden being the primary user [49]. Oats are also often used for human and animal consumption, at least in Scandinavian countries. The results of this study are, therefore, also of relevance for uptake in humans/animals. Oat has also found many less conventional uses—it has been used as a component of cat litter and biodegradable plastics [50]. Therefore, determining the impact of growing plants on soils containing metal complexes with Na_2_EDTA is a key area of research. This raises the question of what effect Na_2_EDTA has on a crop such as *Avena sativa* L. and on biomass production, as well as on the biochemical and physicochemical properties of the soil in the presence and absence of Cr(III) and Cr(VI).

The aim of the present study was to evaluate how soil contamination with Cr(III) and Cr(VI) in the presence of Na2EDTA affects *Avena sativa* L. biomass, assess the remediation capacity of *Avena sativa* L. based on its tolerance index, translocation factor, and chromium accumulation, and investigate how these chromium species affect the soil enzyme activity and physicochemical properties of soil.

## 2. Results

### 2.1. Effect of Chromium on Avena sativa L. Growth and Development

**Figure 1:**
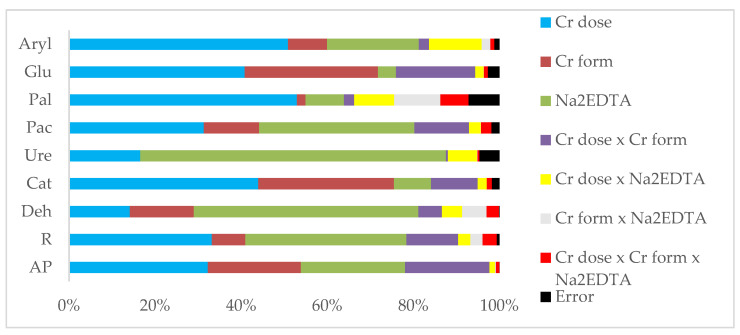
Percentage share of observed variability factors η^2^. Explanations: AP—aboveground parts; R—roots; Deh—dehydrogenases; Cat—catalase; Ure—urease; Pac—acid phosphatase; Pal—alkaline phosphatase; Glu—β-glucosidase; Aryl—arylsulfatase.

Chromium phytotoxicity (expressed as the reduction in biomass yield) varied depending on the soil contamination with Cr, the oxidization state of Cr, and the Na_2_EDTA amendment (Figure 1).

**Figure 2:**
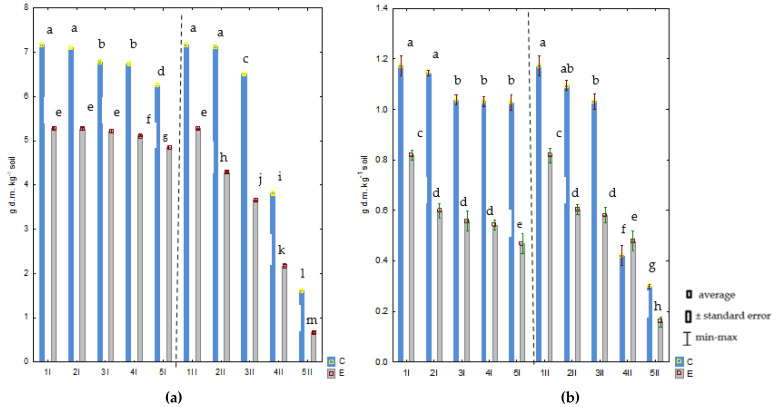
Yield of aboveground parts (**a**) and roots (**b**) of *Avena sativa* L. (g dm kg^−1^ soil) from soil contaminated with chromium (III) and (VI) with Na_2_EDTA. Explanations: 1–0 mg Cr kg^−1^ of soil; 2–5 mg Cr kg^−1^ of soil; 3–10 mg Cr kg^−1^ of soil; 4–20 mg Cr kg^−1^ of soil; 5–40 mg Cr kg^−1^ of soil; I–Cr(III); II–Cr(VI); C—control, E—Na_2_EDTA. Homogeneous groups (a–m) were created separately for aboveground parts and roots.

Cr(III) and Cr(VI) stunted aboveground and root biomass growth in *Avena sativa* L. (Figure 2a,b). The aboveground biomass progressively diminished against the control as the levels of chromium (III) and chromium (VI) in the soil increased. The reduction was more pronounced in the Cr(VI)-contaminated soil than in the Cr(III)-contaminated soil samples. In sites with 40 mg Cr(VI) and Cr(III) kg^−1^ DM of soil, reductions in the biomass of the aboveground parts of *Avena sativa* L. were observed by 78% and 13%, respectively, compared to the uncontaminated sites. On the other hand, the reduction in biomass was higher for roots than for aboveground parts (Figure 1b). The greatest reduction in yield was recorded for Cr(VI) contamination. The root biomass in these objects decreased significantly by 75% compared to the control, while, for chromium (III), it decreased by 12%. Na_2_EDTA, introduced into the soil, caused a reduction in the yield of *Avena sativa* L. (Figure 1a,b). In the series with chromium (VI), a dose of 40 mg Cr(VI) kg^−1^ caused the greatest reductions in the biomass of aboveground parts and roots, by 87% and 81%, respectively, compared to the uncontaminated sites.

**Figure 3:**
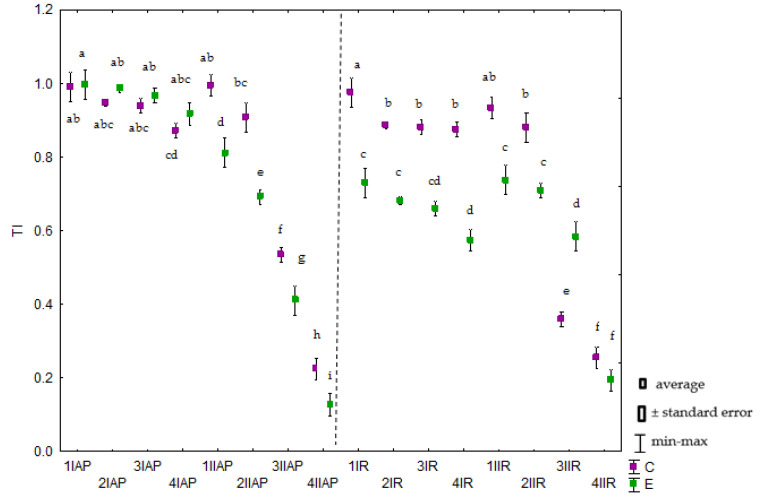
Tolerance index (TI) of *Avena sativa* L. to soil contamination with chromium (III) and (VI). Explanations: 1–0 mg Cr kg^−1^ of soil; 2–5 mg Cr kg^−1^ of soil; 3–10 mg Cr kg^−1^ of soil; 4–20 mg Cr kg^−1^ of soil; 5–40 mg Cr kg^−1^ of soil; I—Cr(III); II—Cr(VI); AP—aboveground parts; R—roots; C—control, E—Na_2_EDTA. Homogeneous groups (a–i) were created separately for aboveground parts and roots.

The tolerance indices (TI) showed that *Avena sativa* L. was more tolerant to Cr(III) contamination than to Cr(VI) contamination. This was particularly noticeable for the highest chromium dose (40 mg kg^−1^). In the no-Na_2_EDTA group, the indices were: 0.871 (aerial parts) and 0.876 (roots) for Cr(III), and 0.224 and 0.254, respectively, for Cr(VI) (Figure 3). In the Na_2_EDTA-amended group, the values were: 0.917 (aerial parts) and 0.574 (roots) for Cr(III), and 0.127 and 0.192, respectively, for Cr(VI).

*Avena sativa* L. (aboveground parts and roots) specimens exposed to Cr(VI) absorbed higher amounts of chromium than than those exposed to Cr(III) (Table 1). In the no-Na_2_EDTA group, the aerial parts of *Avena sativa* L. which were grown on Cr(VI)-contaminated soil contained 6.21 mg kg^−1^ chromium, compared to the 1.66 mg kg^−1^ for Cr(III). The chromium levels in the roots were 45.40 and 41.30 mg kg^−1^, respectively. In the Na_2_EDTA-amended group, the Cr(VI)-contaminated specimens contained 16.30 (aboveground parts) and 86.80 (roots) chromium, compared to the 2.19 and 47.90 mg kg^−1^, respectively, found in the Cr(III) runs. The Cr levels in the soil followed a similar pattern, with higher concentrations found in the Cr(VI)-contaminated soils than in the Cr(III) ones—61.60 and 43.30 mg kg^−1^. Na_2_EDTA induced higher levels of chromium in the soil.

There was a higher chromium content in the soil in the experimental series with Cr(VI) than that with Cr(III). This is due to the greater phytotoxic properties of Cr(VI) than Cr(III). This resulted in a lower uptake of chromium by *Avena sativa* L. from soil in the Cr(VI)-contaminated series than the Cr(III)-contaminated series.

*Avena sativa* L. absorbed more chromium from the soils contaminated with Cr(III) than those contaminated with Cr(VI) (Table 2). Chromium uptake was inhibited by the addition of Na_2_EDTA. The metal mobility in *Avena sativa* L. was determined using the translocation factor (TF), which was calculated from the chromium levels in the aerial parts and roots (Table 2) The Na_2_EDTA-amended group had 12% higher TF values for the Cr(III) plants and 27% higher TF values for the Cr(VI) plants compared to the no-Na_2_EDTA group. The translocation values for Cr(III) were much lower than those for Cr(VI), though they below 1.0 in both cases.

The highest accumulation factor (AF) was observed for *Avena sativa* L. grown with Cr(VI) and Na_2_EDTA, which reached 1.59 (Table 2). AF > 1 was also noted for plants exposed to Cr(III) and Cr(VI) with Na_2_EDTA, as well as Cr(VI) without Na_2_EDTA. Similarly, BF_R_ > 1 was recorded for Cr(III)- and Cr(VI)-contaminated soil with Na_2_EDTA, as well as for the no-Cr(VI)/no-Na_2_EDTA specimens (Table 2). The aerial parts of *Avena sativa* L. showed very low levels of bioaccumulated chromium, whether with or without Na_2_EDTA (Table 2). The highest BF_AG_ (0.25) was observed for the Cr (VI) + Na2EDTA soil.

### 2.2. Effect of Chromium on Biochemical and Physicochemical Parameters of Soil

**Figure 4:**
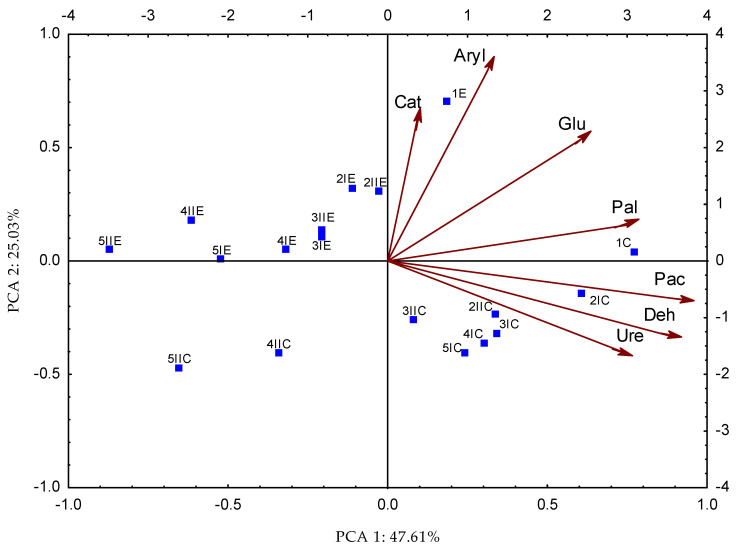
Activity of enzymes in soil contaminated with chromium (III) and (VI) with Na_2_EDTA presented by the PCA method. Explanations: 1–0 mg Cr kg^−1^ of soil; 2–5 mg Cr kg^−1^ of soil; 3–10 mg Cr kg^−1^ of soil; 4–20 mg Cr kg^−1^ of soil; 5–40 mg Cr kg^−1^ of soil; I—Cr(III); II—Cr(VI); C—Control, E—soil with Na_2_EDTA, Deh—dehydrogenases; Cat—catalase; Ure—urease; Pac—acid phosphatase; Pal—alkaline phosphatase; Glu—β-glucosidase; Aryl—arylsulfatase.

In our experiment, the chromium dose accounted for from 14% (dehydrogenases) to 51% (arylsulfatase) of the effect on the enzyme activity, the Cr oxidation state accounted for from 0% (urease) to 31% (β-glucosidase), and the Na2EDTA amendment accounted for from 4% (β-glucosidase) to 71% (urease) (Figure 1). The effect of soil contamination with chromium (III) and (VI) on soil enzyme activity was interpreted using principal component analysis (PCA) (Figure 4). The combined principal components account for 72.64% of the variation in original variables, of which PCA 1 accounted for 47.61%, and PCA 2 accounted for 25.03% (Figure 3). Two homogeneous groups formed around the principal components. The first group comprised catalase, arylsulfatase, β-glucosidase, and alkaline phosphatase vectors, whereas the second comprised acidic phosphatase, dehydrogenases, and urease. The vectors situated along the axes suggest that chromium (III) and (VI) had an adverse effect on soil enzyme activity. The soils that were uncontaminated with Cr(III) and Cr(VI) had the highest rates of enzyme activity, both in the Na_2_EDTA and no-Na_2_EDTA groups. The distribution of the data points relative to the vectors seems to indicate that added Na_2_EDTA not only did not reduce chromium (III) and (VI)-induced stress, but actually exacerbated the adverse effect of Cr on soil enzyme activity.

The values of the index of the chromium effects on soil enzyme activity (IF_Cr_) confirm that chromium had an adverse effect on the biochemical characteristics of soil (Table 3).

Dehydrogenases were found to be the most sensitive to Cr(III) and Cr(VI), whereas catalase proved to be the most resistant. Cr(VI) had more of an inhibitory effect on the tested enzymes than Cr(III). No positive effect of Na2EDTA was observed for the tested enzymes.

Organic carbon content, total nitrogen content, pH, CEC, and BS were mostly unaffected by the chromium species which were tested, remaining fairly stable throughout the study period (Table 4). The hydrolytic acidity increased (except for Cr(VI)-contaminated sites with Na_2_EDTA), and the sum of the base exchangeable cations decreased (except for the site with the highest Cr(III) dose in the series without Na_2_EDTA) under the influence of applied chromium compounds. Soil amendment with Na2EDTA caused higher values of soil pH, but did not significantly alter the other parameters which were studied. In the Cr(III) specimens, the chromium (III) dose was significantly negatively correlated with the activity of catalase, alkaline phosphatase, β-glucosidase, and arylsulfatase, as well as organic carbon content, total nitrogen content, pH, and base saturation (Table 5). In the case of the chromium (VI) specimens, the Cr(VI) dose was significantly negatively correlated with *Avena sativa* L. yield (aerial parts and roots), the activity of all of the tested enzymes, the contents of C_org_ and N_Total_, and the EBC and BS values (Table 6).

## 3. Discussion

### 3.1. Effect of Chromium on Avena sativa L. Growth and Development

Our study found that Cr(III) and Cr(VI) did not disrupt *Avena sativa* L. growth and development at doses of 5 mg kg^−1^ soil, but did result in diminished aerial and root biomass at levels from 10 to 40 mg kg^−1^ d.m. soil. The inhibitory effect of chromium on *Avena sativa* L. was determined by the oxidation state. The 40 mg Cr(VI) and Cr(III) kg^−1^ d.m. soil runs showed a diminished aerial biomass of *Avena sativa* L.—that was 78% and 13% lower than in the non-contaminated specimens, respectively. The decrease for the roots was 75% for Cr(VI) and 12% for Cr (III). Cr(III) is less toxic due to its extremely low solubility, which prevents it from entering groundwater or being taken up by plants. Cervantes et al. [51] found that a Cr(III) dose of 100 mg kg^−1^ caused a 40% reduction in the growth of the aerial parts of barley, whereas Cr(VI) reduced growth by 75% (aerial parts) and 90% (roots). Another study, by Wyszkowska et al. [4], showed that Cr(VI), at 60 mg kg^−1^, reduced the aboveground biomass of *Zea mays* by 90% and the root biomass by 92%. This significant negative effect of Cr (VI) on plants—which also emerged in our study—is caused by disrupted water management, manifested by the wilting and chlorosis of young leaves [47,52]. The reduced biomass is due to the toxic effect of Cr(VI) on photosynthesis and the hindered water/nutrient transport from the soil [53,54]. Stunted root growth may be attributed to the inhibition of root proliferation and elongation, preventing roots from absorbing water and nutrients from the soil [47,55]. Plants take up Cr(III) through a passive mechanism via diffusion at the cell wall cation exchange site [56]. Cr(VI) has structural similarity to phosphate and sulfate, so its uptake occurs through an active process via phosphate and sulfate transporters [57]. The active transport of Cr(VI) results in its immediate conversion to Cr(III) in roots through the action of iron reductase enzymes [58]. This converted Cr(III) binds to the cell wall, thus inhibiting its further transport to the aboveground parts of the plant [59]. In our study, the lower tolerance index (TI) values indicate that Cr(VI) was more toxic to *Avena sativa* L. than Cr(III), and this was further exacerbated by Na_2_EDTA. This is corroborated by Bareen et al. [60], who demonstrated an intensified phytotoxic effect on *Sorghum bicolor* and *Pennisetum glaucum* in specimens treated with both Na_2_EDTA and Cr(VI). The detrimental effects of Na_2_EDTA may be caused by an impaired uptake of essential nutrients, such as Zn^2+^ and Ca^2+^, which in turn decreases cell wall elasticity and viscosity, hampers cell division, disrupts transpiration, and damages cell membranes [61,62]. In the present study, chromium accumulation in the aerial parts and roots was found to be higher in the runs of soil samples contaminated with this metal. According to Rai et al. [53], the chromium concentration in plants will vary depending on the plant species, the metal dose, and the duration of the experiment. For example, *Zea mays* grown on soil contaminated with 10 and 20 mg Cr(VI) kg^−1^ for 30 days contained 15.2 and 16.3 mg Cr kg^−1^ in its aerial parts, respectively [62]. Wyszkowska et al. [4] reported chromium concentrations from 0.66 to 1.04 mg kg^−1^ in the aerial parts of *Zea mays,* and from 1.23 to 17.67 mg kg^−1^ in the roots, when applying doses of 60 mg Cr(VI) kg^−1^. *Cicer arietinum* L. grown on soil contaminated with from 25 to 75 mg Cr(VI) per kg^−1^ soil has been shown to accumulate from 0.0002 to 0.0001 mg chromium kg^−1^ in its roots and from 0.0009 to 0.0005 mg kg^−1^ in its aerial parts [55]. Similarly, Cr accumulation between 0.01 and 0.03 mgkg^−1^ has been demonstrated in *Oryza sativa* L. that has been exposed to 2.5–200 mg kg^−1^ Cr(VI) [63]. In the present study, the aerial parts of *Avena sativa* L. grown on soil contaminated with 40 mg Cr kg^−1^ contained 1.66 mg Cr(III) and 6.21 mg Cr(VI) per kg^−1^, whereas the roots contained 41.30 and 45.40 mg kg^−1^, respectively. The greater accumulation of chromium in *Avena sativa* L. roots, as observed in our study, may be attributed to the reduced transport of chromium from the root to the aerial parts of the plant. The plants may immobilize chromium by compartmentalizing it into the vacuoles, or storing it in the cation exchange sites of the xylem parenchyma cells—a natural defense strategy against metal toxicity [64]. Some smaller proteins act as natural chelates, binding as cations to Cr ions and inhibiting Cr transport [55]. The higher chromium accumulation in the roots may also be explained by the reduction of Cr(VI) to the poorly-soluble Cr(III) [53]. The effect of chromium on *Avena sativa* L. is particularly well demonstrated by the values of the bioaccumulation factors for the aerial parts (BF_AG_) and the roots (BF_R_), as well as by the translocation factor (TF). Bioaccumulation is the ability of plants to neutralize toxic metals into non-toxic or less toxic forms in different plant organs [65]. The BF_AG_ value for *Avena sativa* L. was found to be < 1.0, making the plant a poor candidate for chromium (III) and (VI) phytoextraction. The TF index values were also lower than 1, suggesting that *Avena sativa* L. has a limited capacity to transport chromium from the root to the aerial parts [66,67].

The present study also showed that the addition of Na_2_EDTA increased chromium levels and accumulation in *Avena sativa* L. Na_2_EDTA can bind to chromium to form a Cr-Na_2_EDTA complex or, alternatively, can increase the concentration of the soluble and exchangeable form of Cr by lowering soil pH, thereby increasing bioavailability and promoting transport [68]. In our study, the TF values were higher in Cr(VI)-contaminated samples, and were increased by Na_2_EDTA. Han et al. [69] and Ebrahimi et al. [70] also noted increased Cr accumulation and translocation values in their Cr(III)-contaminated samples, with Na_2_EDTA amendment leading to their further increases in *Phragmites australis* (Cav.) and *Brassica juncea*.

### 3.2. Effect of Chromium on Biochemical and Physicochemical Parameters of Soil

Soil enzymes are synthesized by microorganisms and act as biological catalysts which are involved in metabolic processes that break down organic matter. They reflect the microbial activity in the soil and serve as indicators of metabolic capacity trends in an environment [71,72]. Enzyme tests are considered to be one of the cheapest and easiest techniques for quantifying soil contamination [73,74,75]. The reduction of soluble Cr (VI) to insoluble Cr (III) occurs only in the surface layer of aggregates with higher available organic carbon and higher microbial respiration [76,77]. Therefore, spatial biochemical and microbiological measurements within soil aggregates are needed to characterize and predict the fate of chromium contamination [76]. Soil enzymatic activity is highly sensitive to both natural and anthropogenic disturbances and shows a rapid response to induced changes. Therefore, enzyme activity can be considered an effective indicator of changes in soil quality resulting from environmental stress [78]. The present study found that 5 mg kg^−1^ was the only dose of Cr(III) and Cr(VI) that did not affect enzyme activity to a significant degree. Higher levels of the two metal species inhibited the activity of dehydrogenases, catalase, acidic phosphatase, β-glucosidase, and arylsulfatase, with Cr(VI) being the stronger inhibitor of the two metal species which were tested. Similar findings were reported by Wyszkowska [79], who demonstrated suppressed activity of dehydrogenases, acidic phosphatase, and alkaline phosphatase after exposure to chromium (VI). The results for urease activity were less clear-cut, with Cr(VI) having a stimulating effect at 10 to 40 mg Cr(VI) kg^−1^ soil and an inhibitory effect at the higher doses of 50, 100, and 150 mg Cr(VI) kg^−1^. Of the enzymes that were analyzed, dehydrogenases were found to be the most sensitive to soil contamination with chromium. Dehydrogenases, being intracellular enzymes, occur exclusively in living cells, and the release of heavy metals (including chromium) into the soil can reduce the abundance and activity of reducing/oxidizing microbes [72,80]. Studies by Huang et al. [72] and Peng et al. [81] also demonstrated this sensitivity of dehydrogenases to chromium pollutants. Conversely, catalase proved to be the least sensitive to Cr(III) and Cr(VI) contamination. This is probably due to a reaction between the metal ion in the soil and the functional group of catalase [82,83]. Our findings are corroborated by the results reported by Samborska et al. [84], Al-Khashman and Shawabkeh [85], and Schulin [86]. The chromium-induced inhibition of enzymes may be due to the interaction with the enzyme substrate, denaturation of the enzyme protein, and interaction with its active components [73]. Na_2_EDTA did not eliminate the damaging effects of Cr(III) and Cr(VI) on soil biochemistry—rather, it actually intensified them. The low effectiveness of Na_2_EDTA against chromium may stem from the fact that anionic Cr prevents the formation of a stable complex with Na_2_EDTA. Na_2_EDTA is considered to be the most effective synthetic chelator for the removal of cationic metals, but less so for anionic metals [44,87]. A study by Mahmood-ul-Hassan et al. [88] showed that Cr concentrations were significantly higher in soils enriched with Na_2_EDTA than in soils without its addition. Komárek et al. [89] showed a correlation between soluble Cr concentrations with Na_2_EDTA addition and indicated that dissolved metals persist in contaminated soil even after crop harvest. The use of Na_2_EDTA is problematic, not only because of the excessive mobilization of metals compared to uptake by plants [90], but also because metal complexes with Na_2_EDTA persist for a long time [91], hence the risk of excessive leaching of soluble metals to deeper depths, which can result in the contamination of shallow groundwater [90].

## 4. Materials and Methods

### 4.1. Soil Preparation

Soil samples were taken from the surface layer (0–20 cm deep) in Tomaszkowo near Olsztyn, Warmińsko-Mazurskie Voivodeship, Poland (53.7161° N, 20.4167° E). The soil was crumbled and air-dried, then passed through a 0.5 mm mesh sieve. The choice of soil was primarily dictated by the fact that Poland—which lies in the Central European zone of the subboreal belt and has a temperate climate with oceanic influence—is dominated by zonal soils. These include brown soils, which account for approximately 52% of the country’s area, forming on clay and loam [92]. Prior to the experiment, the soil was analyzed for particle size distribution and basic physicochemical properties (Table 7).

### 4.2. Experimental Procedure

The experiment was conducted in 3.5 kg plastic pots in a greenhouse and consisted of 20 runs in four replications each. The experiment was divided into two groups: non-amended and amended with 1.5 g Na_2_EDTA (di-Sodium versenate dihydrate pure p. a., producer POL-AURA, Morąg, Poland) per kg^−1^ soil. For each run, 3.5 kg soil was weighed and contaminated with (depending on the run): Cr(III) as KCr(SO_4_)_2_.12H_2_O and Cr(VI) as K_2_Cr_2_O_7_ at 5, 10, 20, and 40 mg Cr kg^−1^. Soils uncontaminated with Cr(III) and Cr(VI) served as the control. In 2015, chromium was classified as one of the six pollutants which are highly dangerous to human health [93]. Na_2_EDTA input was set based on Zou et al. [43] and Neugschtner et al. [45]. To provide optimal conditions for *Avena sativa* L. growth and development, all pots were fertilized with the following macro-nutrients: N—140 mg [CO(NH_2_)_2_], P—60 mg [KH_2_PO_4_], K—120 mg [KH_2_PO_4_+KCl], and Mg—20 mg [MgSO_4_∙7H_2_O]. All components (Cr(III) and Cr(VI), Na_2_EDTA, and the fertilizers) were thoroughly mixed with the soil and brought to a moisture content of 50% capillary water capacity. The thus-prepared soil was then potted and sown with the *Avena sativa* L. cultivar ‘Bingo’ (12 plants per pot). Day time ranged from 13 h, 5 min to 16 h, 51 min. The average air temperature was 16.6 C and air humidity was 77.5%. The experiment lasted for 60 days.

### 4.3. Assessment of Plant Growth Performance

Once *Avena sativa* L. was harvested (BBCH 61—beginning of flowering), the dry mass yield of aboveground parts and roots was measured. Chromium was quantified in the aerial (aboveground) parts and roots with ICP-OES (N) (inductively coupled plasma optical emission spectrometry) in Thermo Scientific iCAP 7400 Duo with a TELEDYNE CETAC ASX-560 autosampler (Thermo Scientific, Waltham, MA, USA) according to PN-ISO-11466:2002 [94], after microwave mineralization with 3:1 concentrated nitric acid (V)/hydrogen peroxide.

### 4.4. Biochemical Determinations

Once *Avena sativa* L. was harvested, the soil samples (passed through a 2 mm mesh sieve) were tested for the activity of dehydrogenases [EC 1.1] (according to the procedure provided by Öhlinger [95]), as well as catalase [EC1.11.1.6], urease [EC 3.5.1.5], acid phosphatase [EC 3.1.3.2], alkaline phosphatase [EC 3.1.3.1], β-glucosidase [EC 3.2.1.21], and arylsulfatase [EC 3.1.6.1] (according to Alef and Nannpieri [96]). Extinction of enzymatic reaction products was measured by a PerkinElmer Lambda 25 spectrophotometer (Peabody, MA, USA). Biochemical determinations were performed in triplicate. The protocol used for the enzyme activity assay is detailed in Zaborowska et al. [97] and Borowik et al. [98].

### 4.5. Physicochemical and Chemical Tests

The soil samples were tested for soil pH hydrolytic acidity (HAC), sum of exchangeable base cations (EBC), organic carbon (C_org_), total nitrogen (N_total_), total cation-exchange capacity (CEC), and base saturation (BS). The test protocol is provided in our previous publications [99,100]. Chromium content of the soil was assayed in non-contaminated pots and those contaminated with 40 mg Cr per kg^−1^ dm soil, after microwave mineralization in an extract of 1:3 concentrated nitric acid (V)/concentrated hydrochloric acid (aqua regia). The assay was done by means of ICP-OES according to PN-ISO 11047:2001(A) [101].

### 4.6. Calculations and Statistics

Chromium uptake, tolerance index, translocation factor, bioaccumulation factors, and accumulation factor were calculated from *Avena sativa* L. biomass (aboveground parts and roots) and the plant/soil levels of chromium. Index of chromium effect on soil enzyme activity was also calculated. The index computation methods are detailed in our previous papers [4,102].

The results were statistically processed by analysis of variance (ANOVA) at *p* ≤ 0.05, using STATISTICA 13.1 [103]. Homogeneous groups were generated using Tukey’s test for the following variables: yield of *Avena sativa* L. (aboveground parts and roots), Cr(III) and Cr(VI) in plants and soil, and indices of phytoremediation capacity. Applying multivariate exploratory techniques using Statistica 13.1 software [84], enzyme activity in soil contaminated with Cr(III) and Cr(VI) and with the addition of Na2EDTA was analyzed using principal component analysis—PCA. In turn, the analysis of variance (ANOVA) was used to calculate the coefficient of variation (%) for all considered variables (η^2^). The Pearson linear correlation coefficient was also calculated for the variables.

## 5. Conclusions

Chromium(VI) caused a greater reduction in the aerial and root biomass of *Avena sativa* L. compared with Cr(III). The tolerance indices (TI) showed that *Avena sativa* L. was observed to be more tolerant to Cr(III) contamination than Cr(VI) contamination. The translocation value which was recorded for Cr(III) was much lower than for Cr(VI), though it was at TF < 1 in both cases. Judging by the BF_AG_ < 1, the species does not seem to be suited for chromium (III) and (VI) phytoextraction. Dehydrogenases were found to be the enzymes which were the most sensitive to soil contamination with Cr(III) and Cr(VI). Conversely, catalase was the least sensitive. At 5 mg kg^−1^, the two chromium species did not affect enzyme activity to a significant degree. However, the higher doses of 10, 20, and 40 mg Cr(III) and Cr(VI) kg^−1^ reduced the yields and soil enzyme activity. Na_2_EDTA not only did not reduce Cr(III)- and Cr(VI)-induced stress, but actually augmented the adverse effect of Cr on *Avena sativa* L. and soil enzyme activity.

## Figures and Tables

**Table 1:** The content of chromium in *Avena sativa* L. and soil in mg kg^−1^ d.m.

Cr Dose mg kg^−1^ d.m. Soil	Aboveground Parts		Roots		Soil	
	Cr(III)	Cr(VI)	Cr(III)	Cr(VI)	Cr(III)	Cr(VI)
Control						
0	1.22 ^f^	1.34 ^e^	12.90 ^h^	25.30 ^f^	19.30 ^g^	20.10 ^e^
40	1.66 ^d^	6.21 ^b^	41.30 ^d^	45.40 ^c^	43.30 ^d^	61.50 ^b^
Na_2_EDTA						
0	0.88 ^g^	0.50 ^g^	28.40 ^e^	24.20 ^g^	17.60 ^h^	19.20 ^f^
40	2.19 ^c^	16.30 ^a^	47.90 ^b^	86.80 ^a^	44.10 ^c^	64.90 ^a^

Homogeneous groups (a–h) were created separately for aboveground parts, roots, and soil.

**Table 2:** Uptake (D) chromium by *Avena sativa* L. and indices of translocation (TF) chromium, accumulation (AF), bioaccumulation index in aboveground parts (BF_AG_), bioaccumulation index in roots (BF_R_).

Cr dose mg kg^−1^ d.m. Soil	D µg kg^−1^		TF		AF		BF_AG_		BF_R_	
	Cr(III)	Cr(VI)	Cr(III)	Cr(VI)	Cr(III)	Cr(VI)	Cr(VI)	Cr(III)	Cr(VI)	Cr(VI)
Control										
0	23.86 ^f^	39.25 ^b^	0.10 ^c^	0.05 ^d^	0.73 ^f^	1.33 ^b^	0.06 ^b^	0.07 ^b^	0.67 ^f^	1.26 ^c^
40	52.73 ^a^	23.48 ^g^	0.04 ^e^	0.14 ^b^	0.99 ^d^	0.84 ^e^	0.04 ^b^	0.10 ^b^	0.95 ^e^	0.74 ^f^
Na_2_EDTA										
0	27.86 ^d^	22.42 ^h^	0.03 ^f^	0.02 ^g^	1.66 ^a^	1.29 ^b^	0.05 ^b^	0.03 ^b^	1.61 ^a^	1.26 ^bc^
40	33.06 ^c^	24.54 ^e^	0.05 ^de^	0.19 ^a^	1.14 ^c^	1.59 ^a^	0.05 ^b^	0.25 ^a^	1.09 ^d^	1.34 ^b^

Homogeneous groups (a–h) were created separately for each coefficient.

**Table 3:** Index of the effect of chromium (III) and (VI) with Na2EDTA on enzyme activity.

**(a)**												
**Cr Dose** **mg kg^−1^ d.m. Soil**	**Dehydrogenases**				**Catalase**				**Urease**			
	**Cr(III)**		**Cr(VI)**		**Cr(III)**		**Cr(VI)**		**Cr(III)**		Cr(VI)	
Control												
5	−0.01 ^a^		−0.37 ^i^		−0.02 ^a^		−0.01 ^a^		−0.04 ^a^		−0.04 ^a^	
10	−0.15 ^c^		−0.49 ^j^		−0.03 ^a^		−0.01 ^a^		−0.03 ^a^		−0.03 ^a^	
20	−0.16 ^d^		−0.88 ^o^		−0.04 ^a^		−0.02 ^a^		−0.03 ^a^		−0.03 ^a^	
40	−0.17 ^e^		−0.95 ^p^		−0.04 ^a^		−0.02 ^a^		−0.04 ^a^		−0.06 ^ab^	
$\overset{-}{X}$	−0.12 ^A^		−0.67 ^C^		−0.03 ^D^		−0.01 ^B^		−0.04 ^A^		−0.04 ^A^	
Na_2_EDTA												
5	−0.10 ^b^		−0.45 ^k^		−0.01 ^a^		−0.01 ^a^		−0.08 ^abc^		−0.07 ^ab^	
10	−0.23 ^f^		−0.75 ^l^		−0.02 ^a^		−0.01 ^a^		−0.08 ^abc^		−0.04 ^a^	
20	−0.25 ^g^		−0.78 ^m^		−0.03 ^a^		−0.01 ^a^		−0.123 ^bcd^		−0.16 ^be^	
40	−0.35 ^h^		−0.83 ^n^		−0.03 ^a^		−0.01 ^a^		−0.21 ^e^		−0.20 ^e^	
$\overset{-}{X}$	−0.23 ^B^		−0.70 ^C^		−0.02 ^C^		−0.01 ^A^		−0.12 ^B^		−0.12 ^B^	
**(b)**												
**Cr dose** **mg kg^−1^ d.m. Soil**	**Acid Phosphatase**			**Alkaline Phosphatase**			**β-glucosidase**			**Arylsulfatase**		
	**Cr(III)**	**Cr(VI)**		**Cr(III)**		**Cr(VI)**	**Cr(III)**	**Cr(VI)**		**Cr(III)**		**Cr(VI)**
Control												
5	−0.08 ^abc^	−0.02 ^ab^		−0.01 ^bc^		−0.06 ^cd^	−0.01 ^a^	−0.06 ^abc^		−0.01 ^a^		−0.36 ^c^
10	−0.11 ^cd^	−0.18 ^d^		−0.08 ^cd^		−0.10 ^cd^	−0.02 ^a^	−0.06 ^abc^		−0.09 ^ab^		−0.37 ^c^
20	−0.12 ^cd^	−0.35 ^e^		−0.09 ^cd^		−0.11 ^d^	−0.03 ^a^	−0.16 ^cd^		−0.13 ^b^		−0.46 ^cde^
40	−0.13 ^cd^	−0.52 ^f^		−0.11 ^d^		−0.12 ^d^	−0.03 ^a^	−0.23 ^d^		−0.14 ^b^		−0.54 ^e^
$\overset{-}{X}$	−0.11 ^B^	−0.27 ^C^		−0.07 ^C^		−0.10 ^D^	−0.02 ^A^	−0.13 ^C^		−0.09 ^A^		−0.43 ^B^
Na_2_EDTA												
5	−0.01 ^a^	−0.10 ^bcd^		−0.04 ^bce^		0.07 ^a^	−0.02 ^a^	−0.06 ^abc^		−0.37 ^c^		−0.48 ^de^
10	−0.02 ^a^	−0.13 ^cd^		−0.05 ^cd^		0.04 ^ab^	−0.05 ^ab^	−0.15 ^bce^		−0.41 ^cd^		−0.50 ^de^
20	−0.08 ^abc^	−0.37 ^e^		−0.06 ^cd^		−0.02 ^bc^	−0.05 ^ab^	−0.16 ^bce^		−0.45 ^cde^		−0.51 ^de^
40	−0.10 ^bcd^	−0.37 ^e^		−0.09 ^cd^		−0.07 ^cd^	−0.06 ^abc^	−0.24 ^d^		−0.50 ^de^		−0.52 ^e^
$\overset{-}{X}$	−0.05 ^A^	−0.24 ^C^		−0.05 ^B^		0.01 ^A^	−0.05 ^B^	−0.15 ^D^		−0.43 ^B^		−0.50 ^C^

Homogeneous groups (a–n) were generated separately for each enzyme; homogeneous groups for means were calculated separately for each enzyme (A–D).

**Table 4:** Physicochemical properties of soil contaminated with chromium (III) and (VI) with Na_2_EDTA.

Cr Dose mg kg^−1^ d.m. Soil	C_org_		N_Total_		pH_KCl_		HAC		EBC		CEC		BS%	
	%						(mmol^(+)^ kg^−1^ Soil)							
	Cr(III)	Cr(VI)	Cr(III)	Cr(VI)	Cr(III)	Cr(VI)	Cr(III)	Cr(VI)	Cr(III)	Cr(VI)	Cr(III)	Cr(VI)	Cr(III)	Cr(VI)
Control														
0	0.72 ^a^	0.72 ^a^	0.13 ^ab^	0.13 ^ab^	6.10 ^c^	6.10 ^c^	9.19 ^g^	9.19 ^g^	33.00 ^e^	33.00 ^e^	42.19 ^e^	42.19 ^e^	78.21 ^ef^	78.21 ^ef^
5	0.70 ^a^	0.65 ^cd^	0.13 ^ab^	0.12 ^bc^	5.95 ^cd^	5.95 ^cd^	9.19 ^g^	9.75 ^def^	37.00 ^d^	42.00 ^a^	46.19 ^d^	51.75 ^a^	80.10 ^bcd^	81.16 ^bc^
10	0.66 ^bc^	0.65 ^cd^	0.13 ^ab^	0.10 ^ef^	5.95 ^cd^	5.90 ^d^	9.94 ^de^	10.50 ^c^	33.00 ^e^	41.00 ^ab^	42.94 ^e^	51.50 ^a^	76.85 ^fg^	79.610 ^cde^
20	0.60 ^e^	0.64 ^cd^	0.12 ^bcd^	0.10 ^ef^	5.85 ^de^	5.90 ^d^	10.13 ^cd^	11.25 ^b^	31.00 ^e^	37.00 ^d^	41.13 ^e^	48.25 ^bcd^	75.37 ^g^	76.68 ^fg^
40	0.60 ^e^	0.64 ^d^	0.12 ^bcd^	0.09 ^f^	5.70 ^e^	5.90 ^d^	10.50 ^c^	11.44 ^b^	40.00 ^abc^	32.00 ^c^	50.50 ^ab^	43.44 ^e^	79.21 ^de^	73.67 ^h^
$\overset{-}{X}$	0.66 ^A^	0.66 ^A^	0.12 ^A^	0.11 ^B^	5.91 ^B^	5.95 ^B^	9.79 ^C^	10.43 ^B^	37.00 ^B^	39.20 ^A^	46.79 ^B^	49.63 ^A^	78.85 ^B^	78.77 ^B^
r	−0.89	−0.63	−0.96	−0.90	−0.97	−0.64	0.91	0.90	−0.12	−0.99	−0.02	−0.99	−0.38	−0.98
Na_2_EDTA														
0	0.71 ^a^	0.71 ^a^	0.14 ^a^	0.14 ^a^	6.70 ^a^	6.70 ^a^	9.56 ^efg^	9.56 ^efg^	40.00 ^abc^	40.00 ^abc^	49.56 ^abc^	49.56 ^abc^	80.71 ^bcd^	80.71 ^bcd^
5	0.687 ^b^	0.68 ^b^	0.14 ^a^	0.11 ^cde^	6.65 ^a^	6.65 ^a^	9.94 ^de^	8.06 ^i^	33.00 ^e^	40.00 ^abc^	42.94 ^e^	48.06 ^cd^	76.84 ^fg^	83.23 ^a^
10	0.68 ^b^	0.68 ^b^	0.13 ^ab^	0.11 ^cde^	6.60 ^a^	6.65 ^a^	11.63 ^ab^	8.63 ^h^	31.00 ^e^	38.00 ^cd^	42.63 ^e^	46.63 ^d^	72.72 ^h^	81.50 ^b^
20	0.67 ^b^	0.67 ^b^	0.13 ^ab^	0.11 ^de^	6.35 ^b^	6.55 ^a^	12.00 ^a^	9.19 ^g^	31.00 ^e^	39.00 ^bcd^	43.00 ^e^	48.19 ^bcd^	72.08 ^h^	80.93 ^bc^
40	0.67 ^b^	0.66 ^bc^	0.12 ^bc^	0.11 ^de^	5.95 ^cd^	6.55 ^a^	12.00 ^a^	9.38 ^fg^	31.00 ^e^	32.00 ^e^	43.00 ^e^	41.38 ^e^	72.08 ^h^	77.34 ^f^
$\overset{-}{X}$	0.68 ^A^	0.68 ^A^	0.13 ^A^	0.12 ^B^	6.45 ^A^	6.62 ^A^	11.03 ^A^	8.55 ^D^	33.20 ^C^	37.80 ^B^	44.23 ^C^	46.35 ^B^	74.89 ^C^	81.44 ^A^
r	−0.68	−0.84	−0.88	−0.60	−0.99	−0.88	0.79	0.89	−0.63	−0.92	−0.51	−0.84	−0.75	−0.99

C_org—_total organic carbon; N_total_—total nitrogen; HAC—hydrolytic acidity; EBC—total exchangeable cations; CEC—total exchange capacity of soil; BS—basic cations saturation ratio in soil. Homogeneous groups (a–i) were created separately for each parameter; homogeneous groups for means were calculated separately for each parameter (A–D); r—correlation coefficient significant at *p* = 0.05, *n* = 16.

**Table 5:** Coefficients of correlation between variables in soil contaminated with chromium (III).

Variable Factors	AP	R	Deh	Cat	Ure	Pac	Pal	Glu	Aryl	C_org_	N_Total_	pH	HAC	EBC	CEC	BS
Dose Cr	−0.26	−0.31	−0.15	−0.85 *	−0.35	−0.32	−0.51 *	−0.63 *	−0.65 *	−0.76 *	−0.72 *	−0.55 *	0.71 *	−0.24	−0.03	−0.56 *
AP	1.00	0.96 *	0.98 *	−0.04	0.88 *	0.96 *	0.58 *	−0.48 *	−0.27	−0.07	−0.21	−0.60 *	−0.72 *	0.18	−0.04	0.54 *
R		1.00	0.96 *	0.05	0.93 *	0.94 *	0.58 *	−0.38 *	−0.06	−0.07	−0.15	−0.55 *	−0.80 *	0.42 *	0.21	0.72 *
Deh			1.00	−0.11	0.88 *	0.96 *	0.55 *	−0.55 *	−0.32	−0.16	−0.31	−0.69 *	−0.69 *	0.27	0.07	0.57 *
Cat				1.00	0.05	0.07	0.17	0.77 *	0.69 *	0.72 *	0.67 *	0.65 *	−0.48 *	0.20	0.07	0.40 *
Ure					1.00	0.89 *	0.68 *	−0.33	−0.00	−0.12	−0.06	−0.42 *	−0.77 *	0.41 *	0.20	0.69 *
Pac						1.00	0.63 *	−0.41 *	−0.236	−0.03	−0.16	−0.54 *	−0.74 *	0.27	0.06	0.60 *
Pal							1.00	−0.04	0.08	0.34	0.19	−0.150	−0.59 *	0.25	0.09	0.49 *
Glu								1.00	0.82 *	0.60 *	0.70 *	0.86 *	−0.15	0.22	0.19	0.20
Aryl									1.00	0.47 *	0.63 *	0.68 *	−0.34	0.49 *	0.44 *	0.46 *
C_org_										1.00	0.63 *	0.53 *	−0.26	0.01	−0.08	0.16
N_Total_											1.00	0.69 *	−0.31	0.14	0.06	0.27
pH												1.00	0.07	−0.05	−0.03	−0.08
HAC													1.00	−0.50 *	−0.23	−0.88 *
EBC														1.00	0.96 *	0.85 *
CEC															1.00	0.67 *

C_org_—total organic carbon, N_total_—total nitrogen, HAC—hydrolytic acidity, EBC—total exchangeable cations, CEC—total exchange capacity of soil, BS—basic cations saturation ratio in soil; AP—yield aboveground parts; R—yield roots^;^ Deh—dehydrogenases; Cat—catalase; Ure—urease; Pac—acid phosphatase; Pal—alkaline phosphatase; Glu—β-glucosidase; Aryl—arylsulfatase; * r—coefficient of correlation significant at: *p* = 0.05, *n* = 30.

**Table 6:** Coefficients of correlation between variables in soil contaminated with chromium (VI).

Variable Factors	AP	R	Deh	Cat	Ure	Pac	Pal	Glu	Aryl	C_org_	N_Total_	pH	HAC	EBC	CEC	BS
Dose Cr	−0.84 *	−0.82 *	−0.68 *	−0.55 *	−0.43 *	−0.79 *	−0.64 *	−0.94 *	−0.64 *	−0.71 *	−0.84 *	−0.18	0.46 *	−0.47 *	−0.35	−0.59 *
AP	1.00	0.97 *	0.86 *	0.21	0.73 *	0.96 *	0.51 *	0.74 *	0.21	0.33	0.58 *	−0.33	−0.10	0.52 *	0.51 *	0.39 *
R		1.00	0.89 *	0.27	0.65 *	0.94 *	0.51 *	0.75 *	0.24	0.40 *	0.61 *	−0.28	−0.15	0.48 *	0.45 *	0.39 *
Deh			1.00	0.22	0.64 *	0.91 *	0.45 *	0.55 *	0.12	0.43 *	0.54 *	−0.42 *	−0.06	0.09	0.08	0.09
Cat				1.00	−0.25	0.26	0.54 *	0.59 *	0.62 *	0.68 *	0.61 *	0.65 *	−0.69 *	0.14	−0.06	0.54 *
Ure					1.00	0.71 *	0.28	0.26	−0.15	−0.02	0.13	−0.73 *	0.49 *	0.12	0.27	−0.24
Pac						1.00	0.48 *	0.65 *	0.16	0.33	0.60 *	−0.36 *	−0.09	0.37 *	0.35	0.28
Pal							1.00	0.62 *	0.22	0.54 *	0.41 *	0.17	−0.48 *	0.20	0.06	0.44 *
Glu								1.00	0.71 *	0.71 *	0.82 *	0.32	−0.51 *	0.54	0.41 *	0.67 *
Aryl									1.00	0.78 *	0.82 *	0.66 *	−0.46 *	0.19	0.06	0.42 *
C_org_										1.00	0.75 *	0.52 *	−0.60 *	−0.04	−0.22	0.37 *
N_Total_											1.00	0.37 *	−0.47 *	0.29	0.16	0.48 *
pH												1.00	−0.78 *	0.11	−0.12	0.57 *
HAC													1.00	−0.25	0.04	−0.80
EBC														1.00	0.96 *	0.77 *
CEC															1.00	0.56 *

C_org_—total organic carbon, N_total_—total nitrogen, HAC—hydrolytic acidity, EBC—total exchangeable cations, CEC—total exchange capacity of soil, BS—basic cations saturation ratio in soil; AP—yield aboveground parts; R—yield roots; Deh—dehydrogenases; Cat—catalase; Ure—urease; Pac—acid phosphatase; Pal—alkaline phosphatase; Glu—β-glucosidase; Aryl—arylsulfatase; * r—coefficient of correlation significant at: *p* = 0.05, *n* = 30.

**Table 7:** Some physicochemical properties of the soil used in the experiment.

Type of Soil	Granulometric Composition (%)			pH_KCl_	C_org_	N_total_	HAC	EBC	CEC	BS%
	Sand	Silt	Clay		g kg^−1^		mmol^(+)^ kg^−1^ Soil			
ls	69.41	27.71	2.88	6.09	6.18	1.27	8.81	24.00	32.81	73.14

ls—sandy loam, C_org—_total organic carbon, N_total_—total nitrogen, HAC—hydrolytic acidity, EBC—total exchangeable cations, CEC—total exchange capacity of soil, BS—basic cations saturation ratio in soil.

## Data Availability

Not applicable.

## Sample Availability

Samples of the compound are not available from the authors.
